# Efficient Remyelination Requires DNA Methylation

**DOI:** 10.1523/ENEURO.0336-16.2017

**Published:** 2017-03-30

**Authors:** Sarah Moyon, Dan Ma, Jimmy L. Huynh, David J.C. Coutts, Chao Zhao, Patrizia Casaccia, Robin J.M. Franklin

**Affiliations:** 1 Department of Neuroscience, Icahn School of Medicine at Mount Sinai, New York, NY 10029; 2 Department of Genetics and Genomic Sciences, Icahn School of Medicine at Mount Sinai, New York, NY 10029; 3 Neuroscience Initiative Advanced Science Research Center, CUNY, New York, NY 10031; 4 Wellcome Trust-Medical Research Council Cambridge Stem Cell Institute and Department of Clinical Neurosciences, University of Cambridge, Cambridge, CB2 0AH, UK

**Keywords:** Adult oligodendrocyte progenitor cells, DNA methylation, remyelination

## Abstract

Oligodendrocyte progenitor cells (OPCs) are the principal source of new myelin in the central nervous system. A better understanding of how they mature into myelin-forming cells is of high relevance for remyelination. It has recently been demonstrated that during developmental myelination, the DNA methyltransferase 1 (DNMT1), but not DNMT3A, is critical for regulating proliferation and differentiation of OPCs into myelinating oligodendrocytes (OLs). However, it remains to be determined whether DNA methylation is also critical for the differentiation of adult OPCs during remyelination. After lysolecithin-induced demyelination in the ventrolateral spinal cord white matter of adult mice of either sex, we detected increased levels of DNA methylation and higher expression levels of the DNA methyltransferase DNMT3A and lower levels of DNMT1 in differentiating adult OLs. To functionally assess the role of DNMT1 and DNMT3 in adult OPCs, we used mice with inducible and lineage-specific ablation of *Dnmt3a* and/or *Dnmt1* (i.e., *Plp-creER(t);Dnmt3a-flox*, *Plp-creER(t);Dnmt1-flox, Plp-creER(t);Dnmt1-flox;Dnmt3a-flox*). Upon lysolecithin injection in the spinal cord of these transgenic mice, we detected defective OPC differentiation and inefficient remyelination in the *Dnmt3a* null and *Dnmt1/Dnmt3a* null mice, but not in the *Dnmt1* null mice. Taken together with previous results in the developing spinal cord, these data suggest an age-dependent role of distinct DNA methyltransferases in the oligodendrocyte lineage, with a dominant role for DNMT1 in neonatal OPCs and for DNMT3A in adult OPCs.

## Visual Abstract

**Figure:**
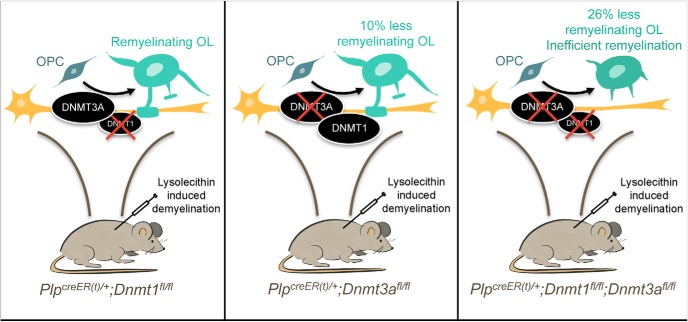

## Significance Statement

The regenerative therapy of enhancing remyelination is the subject of much current investigation for a number of central nervous system disorders. However, its mechanisms remain incompletely understood. A recent study identified a distinct role of the DNA methyltransferase 1 (DNMT1) in developmental myelination; here we report a dominant role for DNMT3A in adult remyelination after lysolecithin-induced demyelination. Overall, this is of high relevance, as it indicates that neonatal and adult oligodendrocyte progenitor cells might be characterized by distinct epigenetic landscapes that may need to be taken into consideration for the development of future therapeutic strategies.

## Introduction

In demyelinating disorders, such as multiple sclerosis (MS), loss of myelin sheaths disturbs axonal conduction and trophic support, eventually leading to irreversible axonal loss and disease progression (Trapp et al. 1998; Nave and Trapp, 2008; Franklin et al. 2012). Remyelination, which restores myelin sheaths to demyelinated axons and thereby restores both axonal function and protection, is regarded as a promising way to prevent disease progression (Dubois-Dalcq et al. 2008; Franklin and Ffrench-Constant, 2008). Oligodendrocyte progenitor cells (OPCs) have been identified as the main source for new myelin formation in the adult central nervous system (CNS; Zawadzka et al. 2010). Therefore, a better understanding of the molecular mechanism regulating their differentiation into myelin-forming cells is highly desirable. It has been proposed that after demyelination, adult OPC differentiation recapitulates developmental myelination to a large extent, and the expression of well-established differentiation regulatory transcription factors (e.g., *Myrf*, *Nkx2.2*, *Tcf4*, *Sox2*) has been shown to change during remyelination (Fancy et al. 2004, 2009; Koenning et al. 2012; Moyon et al. 2015; Zhao et al. 2015).

The importance of posttranslational histone modifications during remyelination has previously been reported (Shen et al. 2008). Recently, it has been shown that DNA methylation mediated by the DNA methyltransferase DNMT1 is essential for developmental myelination, where it controls the transition from the proliferative OPC stage to differentiating oligodendrocytes (OLs; Moyon et al. 2016). In this study, we asked whether similar epigenetic mechanisms might be involved in the regulation of adult OPC differentiation during remyelination. Transcriptomic data gathered from laser microdissected regions of CNS white matter at various times after acute experimentally induced demyelination indicate that the expression of *Dnmt1* and *Dnmt3a* are differentially regulated during remyelination (Huang et al. 2011). Both enzyme levels were higher at 5 days post-lesion (dpl), during the early stages of remyelination, and lower at 14 and 28 dpl, suggesting that DNA methylation might also play a role in the transition from adult OPCs to myelinating OLs. A recent study has previously reported genome-wide DNA methylation changes in postmortem brain samples from MS patients compared with controls, suggesting an underlying dysregulation of DNA methylation in MS brains (Huynh et al. 2014).

This study directly addresses the role of DNA methylation in oligodendroglial lineage cells during remyelination in the adult spinal cord. Here we show that DNA methylation and DNA methyltransferase levels are differentially regulated during remyelination. We use lineage-specific inducible genetic ablation of *Dnmt1* or *Dnmt3a* in adult mice to address the functional relevance of DNA methylation perturbations for adult OPC differentiation and the efficiency of remyelination after experimentally induced demyelination.

## Materials and Methods

### Animals

All experiments were performed according to institutional animal care and use committee–approved protocols and mice were maintained in a temperature- and humidity-controlled facility on a 12-h light-dark cycle with food and water ad libitum. *Dnmt1^fl/fl^* (Fan et al. 2001; Jackson-Grusby et al. 2001, RRID:MMRRC_014114-UCD) and *Dnmt3a^fl/fl^* (Kaneda et al. 2004, RRID:MGI:3718448) mice on a C57BL/6 background were crossed with *Plp-creER(t)* (The Jackson Laboratory, RRID:MGI:3696409; Doerflinger et al. 2003).

### Lysolecithin injections

Injections were conducted in the ventrolateral spinal cord white matter of 8-week-old animals of either sex, as previously described (Fancy et al. 2009). Briefly, anesthesia was induced and maintained with inhalational isoflurane/oxygen. The vertebral column was fixed between metal bars on stereotaxic apparatus. The spinal vertebra was exposed, tissue was cleared overlying the intervertebral space, and the dura was pierced. A pulled-glass needle was advanced through the spine, at an angle of 70°, and 1 μl of 1% lysolecithin (Sigma-Aldrich L4129) was slowly injected into the ventrolateral white matter. Mice were sutured and kept in a warm chamber during recovery.

### Tamoxifen injections

4-Hydroxytamoxifen (Sigma-Aldrich T56-48) was dissolved at 40 mg/ml in 10% ethanol and 90% corn oil (Sigma-Aldrich C8267) for 4 h at 37°C with rotation, and 10 mg was administered by gavage to each mouse at days 3, 5, and 7 (for 14 dpl analysis) or at days 5, 7, and 9 (21 dpl analysis) after lysolecithin injection (day 0).

### Immunohistochemistry

For immunohistochemistry, animals were perfused at 5, 14, or 21 dpl with 4% paraformaldehyde and postfixed overnight in the same solution at 4°C. Spinal cords were dissected, cryoprotected in sucrose solutions, and frozen embedded in OCT. Immunohistochemistry was performed on 12-μm cryostat sections. Antigen retrieval was performed for 5-methylcytosine (5mC) staining by incubating slides in subboiling (94°C) citrate buffer (pH 6.0) for 15 min. Slides were incubated in blocking buffer (5% normal donkey serum in PBS/Triton X-100 0.3%) for 1 h at room temperature and then overnight at 4°C with the primary antibodies diluted in a similar blocking buffer (5% normal donkey serum in PBS/Triton X-100 0.3%). After rinsing with PBS 1×, sections were incubated with the Alexa Fluor secondary antibodies and then washed with PBS 1×. Cell nuclei were counterstained with DNA fluorescent dye Hoechst 33342 (Sigma B2261) in PBS. Stained tissue or cells were coverslipped in FluorSave mounting medium (Millipore 345789) and examined on a Zeiss Axio Observer fluorescence microscope. To quantify the data generated by immunohistochemical staining, counts were undertaken by an observer who was blinded to the experimental group from which the sample being analyzed was taken. Counts were made throughout the entire lesion area which was scanned using the 20× objective of the fluorescence microscope. Labeled cells were manually counted from the images captured under the same exposure conditions. AxioVision Rel4.8 software (RRID:SCR_002677) was used for colocalized color identification and area measurement. Quantification of total cell number, as defined by nuclear (DAPI) staining, was assessed both within the lesion area and within the corresponding region of white matter in unlesioned tissue.

To assess the levels of 5mC in OLIG2^+^ cells, arbitrarily defined as being either low, medium, or high, a macro was created in ImageJ (RRID:SCR_003070) that first localized the OLIG2^+^ (red) nuclei and then measured the intensity of the 5mC (green) staining within the nuclear area. The intensity value was then normalized by deducting the background staining intensity. For all quantifications, a minimum of three sections of 12-µm thickness from each lesion randomly chosen from *n* = 4–6 mice was examined. The percentage or density of cells was determined per mouse. The average and standard error was then calculated for each group using GraphPad Prism (GraphPad, RRID:SCR_002798).

### Antibodies

Primary antibodies used are: mouse anti-5mC (Abcam, ab10805, RRID:AB_442823, 1:200), mouse anti-CC1/APC (Millipore OP80, RRID:AB_2057371, 1:300), rabbit anti-DNMT1 (Abcam, ab19905, RRID:AB_731983, 1:1000), rabbit anti-DNMT3A (Santa Cruz, sc-20703, RRID:AB_2093990, 1:500), mouse anti-NKX2.2 (Developmental Studies Hybridoma Bank, University of Iowa, Iowa City, IA, 1:100), and rabbit anti-OLIG2 (Millipore, ab9610, RRID:AB_10141047, 1:1000). Alexa Fluor–conjugated secondary antibodies (1:5000) were used (Invitrogen).

### Electron microscopy

For electron microscopy, animals were perfused at 21 dpl with 4% glutaraldehyde in PBS containing 0.4 mm CaCl_2_ and postfixed in the same solution at 4°C. The spinal cord was coronally sliced at 1-mm thickness and treated with 2% osmium tetroxide overnight before being subjected to a standard protocol for epoxy resin embedding (Zhao et al. 2008) Tissues were sectioned at 1 μm and stained with toluidine blue. Remyelination ranking, in which lesions with the greatest extent of remyelination were assigned the highest rank value, was performed under light microscopy (Ibanez et al. 2003). Ultrathin sections of the lesion site were cut onto copper grids and stained with uranyl acetate before being examined with a Hitachi H-600 transmission electron microscope. G-ratio was quantified on 50-nm sections on a minimum of 70 myelinated and remyelinated axons per animal, three to five mice for each genotype.

### Statistical analysis

All statistical analyses were done using GraphPad Prism (Table 1). Unpaired Student’s *t* test was used for every two datasets with equal variances and for which data followed a normal distribution. If data were not normally distributed, nonparametric Mann–Whitney test was used (for rankings analysis), and if the variances were significantly different, the Welch’s correction was applied (for g-ratio analysis). Two-way ANOVA was used to compare three or more sets of data. For all graphs, error bars are mean ± SEM.

**Table 1.** Statistical analysis

Code	Data structure	Type of test	95% CI
^a^ (Fig. 1C)	Normal distribution, equal variances	ANOVA and Bonferroni posttests	108.6 to 272.7, –27.42 to 136.7 and –73.69 to 90.40
^b^ (Fig. 1D)	Normal distribution, equal variances	ANOVA and Bonferroni posttests	51.11 to 98.17 and 11.76 to 58.82 (5dpl); 33.78 to 80.84 and –11.49 to 35.57 (14dpl); 25.89 to 72.95 and –14.29 to 32.77 (21dpl)
^c^ (Fig. 1F)	Normal distribution, equal variances	ANOVA and Bonferroni posttests	45.42 to 116.2, 176.9 to 247.7 and 133.9 to 204.7
^d^ (Fig. 1G)	Normal distribution, equal variances	ANOVA and Bonferroni posttests	–2.914 to 37.65 and 60.67 to 101.2 (5dpl); –5.602 to 34.96 and 77.53 to 118.1 (14dpl); –13.63 to 26.93 and 53.74 to 94.30 (21dpl)
^e^ (Fig.1I)	Normal distribution, equal variances	ANOVA and Bonferroni posttests	–73.29 to –34.94, –70.07 to –31.72 and –85.01 to –46.66 (low); 19.25 to 57.60, 8.444 to 46.79 and 16.22 to 54.56 (medium); –4.294 to 34.05, 4.099 to 42.45, 11.27 to 49.62 (high)
^f^ (Fig. 2B)	Normal distribution, equal variances	Student’s *t*-test	201.1 to 231.7 (OLIG2^+^), 191.3 to 215.4 (CC1^+^)
^g^ (Fig. 2D)	Normal distribution, equal variances	Student’s *t*-test	210.2 to 205.7 (OLIG2^+^), 203.9 to 189.2 (CC1^+^)
^h^ (Fig. 2F)	Normal distribution, equal variances	Student’s *t*-test	178.2 to 144.9 (OLIG2^+^), 165.7 to 136.2 (CC1^+^)
^i^ (Fig.2H)	Normal distribution, equal variances	ANOVA and Bonferroni posttests	70.83 to 72.29 (low); 15.67 to 13.36 (medium); 13.50 to 14.35 (high)
^j^ (Fig.2I)	Normal distribution, equal variances	ANOVA and Bonferroni posttests	77.96 to 65.61 (low); 15.85 to 23.22 (medium); 6.186 to 11.17 (high)
^k^ (Fig.2J)	Normal distribution, equal variances	ANOVA and Bonferroni posttests	67.91 to 58.45 (low); 21.32 to 31.39 (medium); 10.77 to 10.16 (high)
^l^ (Fig. 3B)	Normal distribution, equal variances	Student’s *t*-test	–307.8 to 246.4 (OLIG2^+^), –249.9 to 105.2 (CC1^+^) and –16.51 to 17.07 (CC1^+^/OLIG2^+^)
^m^ (Fig. 3D)	Normal distribution, equal variances	Student’s *t*-test	–341.1 to 431.1 (OLIG2^+^), 13.87 to 270.7 (CC1^+^) and 2.758 to 17.22 (CC1^+^/OLIG2^+^)
^n^ (Fig. 3F)	Normal distribution, equal variances	Student’s *t*-test	–275.5 to 52.4 (OLIG2^+^), 12.91 to 264.6 (CC1^+^) and 17.64 to 38.55 (CC1^+^/OLIG2^+^)
° (Fig. 3G)	Normal distribution, equal variances	Student’s *t*-test	36.15 to 226.8 (DNMT1) and –136.3 to 15.88 (DNMT3A); –134.3 to –21.63 (DNMT1) and 48.69 to 246.2 (DNMT3A); 51.76 to 317.0 (DNMT1) and 106.9 to 307.9 (DNMT3A)
*^p^* (Fig.4B)	Normal distribution, equal variances	ANOVA and Bonferroni posttests	22.94 to 25.45 (low); 59.72 to 59.83 (medium); 17.33 to 14.72 (high)
^q^ (Fig.4D)	Normal distribution, equal variances	ANOVA and Bonferroni posttests	22.71 to 31.26 (low); 58.99 to 55.91 (medium); 18.30 to 12.83 (high)
^r^ (Fig.4F)	Normal distribution, equal variances	ANOVA and Bonferroni posttests	21.26 to 37.88 (low); 61.43 to 49.48 (medium); 17.31 to 12.64 (high)
^s^ (Fig. 5B)	Nonnormal distribution	Nonparametric Mann Whitney test	2.648 to 11.35 and 1.362 to 7.838
^t^ (Fig. 5C)	Normal distribution, unequal variances	Student’s *t*-test with Welch’s correction	–0.005297 to 0.005700
^u^ (Fig. 5E)	Nonnormal distribution	Nonparametric Mann Whitney test	1.823 to 11.38 and 2.191 to 9.142
^v^ (Fig. 5F)	Normal distribution, unequal variances	Student’s *t*-test with Welch’s correction	–0.001182 to 0.01204
^x^ (Fig. 5H)	Nonnormal distribution	Nonparametric Mann Whitney test	–1.006 to 9.256 and 2.886 to 6.864
^y^ (Fig. 5G)	Normal distribution, unequal variances	Student’s *t*-test with Welch’s correction	–0.01370 to –0.003870

## Results

### DNA methyltransferases are differently expressed in adult OPCs during remyelination

**Figure 1.**
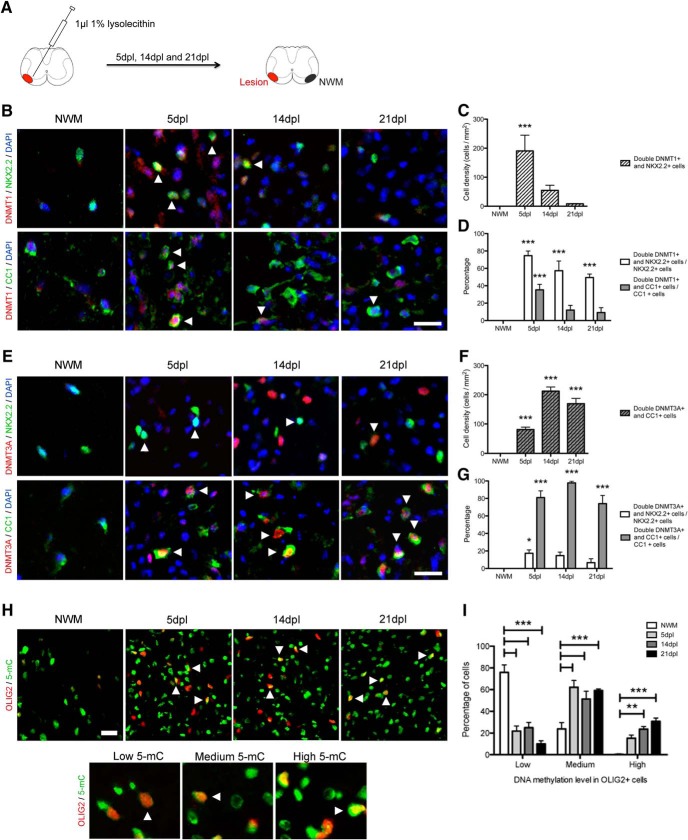
DNA methyltransferases are differently expressed in adult OPCs during remyelination. ***A***, Schematic of the lysolecithin-induced focal demyelination and of the area of NWM used for quantification. ***B***, Representative DNMT1, NKX2.2, and CC1 stainings in NWM and at 5, 14, and 21 dpl (white arrowheads indicate double-positive cells). ***C***, Quantification of the number of double DNMT1^+^ and NKX2.2^+^ cells at 5, 14, and 21 dpl, compared with NWM^a^. ***D***, Quantification of the percentage of double DNMT1^+^ and NKX2.2^+^ or CC1^+^ cells at 5, 14, and 21 dpl, compared with NWM^b^. ***E***, Representative DNMT3A, NKX2.2, and CC1 stainings in NWM and at 5, 14, and 21 dpl (white arrowheads indicate double-positive cells). ***F***, Quantification of the number of double DNMT3A^+^ and CC1^+^ cells at 5, 14, and 21 dpl, compared with NWM^c^. ***G***, Quantification of the percentage of double DNMT3A^+^ and NKX2.2^+^ or CC1^+^ cells at 5, 14, and 21 dpl, compared with NWM^d^. ***H***, Representative 5mC and OLIG2 staining in NWM and at 5, 14, and 21 dpl (white arrowheads indicate high-5mC^+^/OLIG2^+^ cells). Representative low-, medium-, and high-5mC cells are shown below. ***I***, Quantification of low-, medium-, and high-5mC levels in OLIG2^+^ cells at 5, 14, and 21 dpl, compared with NWM^e^. Scale bar = 20 µm. Data are mean ± SEM. *n* = 4–6 animals, three sections per animal. *p < 0.05, **p < 0.01, ***p < 0.001 (ANOVA).

To begin characterizing the role of DNA methylation in the oligodendroglial lineage during remyelination, we performed lysolecithin injections in the ventrolateral spinal cord of 8-week-old C57BL/6 mice and perfused them at 5, 14, and 21 dpl, to access DNMT1, DNMT3A, and 5mC expression in OPCs and OLs (Fig. 1*A*). The number of NKX2.2^+^ oligodendroglial cells strongly expressing DNMT1, while abundant at 5 dpl when compared with levels in surrounding intact nonlesioned white matter (NWM), decreased between 5 and 21 dpl (Fig. 1*B*, *C*). Costaining of DNMT1 with CC1, a marker of mature OLs, or NKX2.2, a marker of OPCs, revealed that within the CC1^+^ population the enzyme was expressed in 35.3 ± 6.3% (5 dpl), 12.0 ± 5.4% (14 dpl), and 9.2 ± 5.6% (21 dpl) of cells, whereas in the NKX2.2^+^ population, it was expressed in 74.6 ± 5.3% (5 dpl), 57.3 ± 11.1% (14 dpl), and 49.4 ± 4.0% (21 dpl) of cells (Fig. 1*B*, *D*). In contrast, the distribution of DNMT3A showed a different pattern, being strongly expressed by CC1^+^ cells and increased from 5 to 14 dpl (Fig. 1*E*, *F*): DNMT3A was expressed in 81.0 ± 7.6% (5 dpl), 97.8 ± 1.9% (14 dpl), and 74.0 ± 9.3% (21dpl) of the CC1^+^ OL population and in only 17.4 ± 3.8% (5 dpl), 14.7 ± 4.0% (14 dpl), and 6.7 ± 4.4% (21 dpl) of the NKX2.2^+^ population (Fig. 1*E*, *G*). These data indicate distinct patterns of expression, with DNMT1 mainly expressed by adult OPCs during the early stages of remyelination, and DNMT3A mainly detected in differentiated adult OLs that appear in the later stages of remyelination.

We also quantified 5mC expression levels in oligodendroglial cells. Because of species similarities in the 5mC antibodies and those we had used for the OL lineage, we combined 5mC staining with the pan-OL lineage marker OLIG2. This revealed a decreased proportion of low-methylated and increased proportion of medium- and high-methylated oligodendroglial cells during remyelination (Fig. 1*H*, *I*). Hypermethylation in OLIG2^+^ cells was already evident starting at 5 dpl (Fig. 1*I*).

### Ablation of DNMT3A and both DNMT1 and 3A impairs oligodendrocyte differentiation during remyelination

To address more specifically the functional role of DNA methylation in adult OPCs during remyelination, we crossed the *Dnmt1^fl/fl^* and *Dnmt3a^fl/fl^* lines with the inducible *Plp-creER(t)*, to target specific ablation of *Dnmt1*, *Dnmt3a*, or both *Dnmt1* and *Dnmt3a* in proteolipid protein (PLP)-expressing oligodendroglial cells after lysolecithin-induced demyelination. All three mutants (*Plp^creER(t)^* ^/^*^+^*:*Dnmt1^fl/fl^*; *Plp^creER(t)^* ^/^*^+^*:*Dnmt3a^fl/fl^*; and *Plp^creER(t)^* ^/^*^+^*:*Dnmt1^fl/fl^;Dnmt3a^fl/fl^*) and control littermates (*Plp^+^* ^/^*^+^*:*Dnmt1^fl/fl^*; *Plp^+^* ^/^*^+^*:*Dnmt3a^fl/fl^* and *Plp^+^* ^/^*^+^:Dnmt1^fl/fl^;Dnmt3a^fl/fl^*) were gavaged with tamoxifen at 3, 5, and 7 dpl and then lesion-containing tissue harvested at 14 dpl and processed for immunohistochemistry using antibodies specific for 5mC, for mature oligodendrocytes (CC1), or for all cells within the oligodendrocyte lineage (OLIG2).

**Figure 2.**
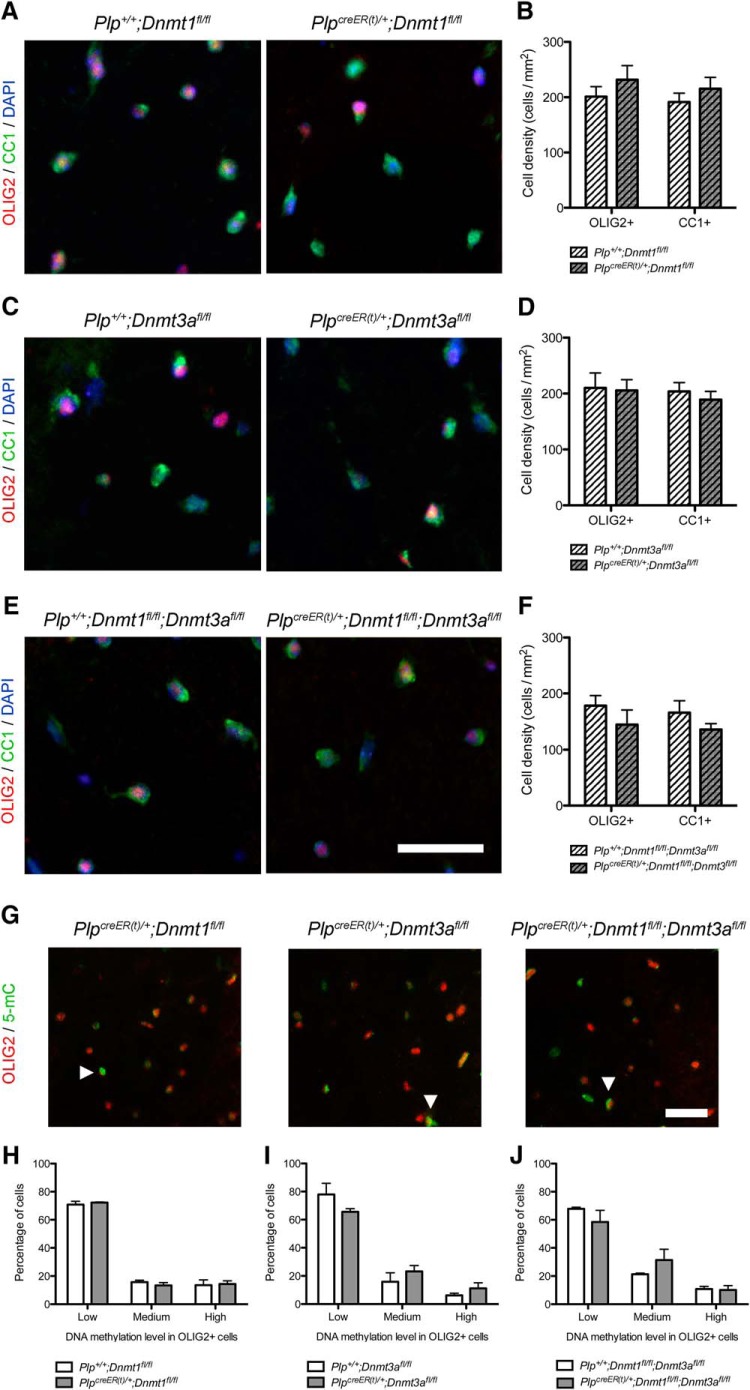
Ablation of *Dnmt1* or *Dnmt3a* does not impair oligodendrocyte differentiation or their methylation levels in control conditions. ***A***, Representative OLIG2 and CC1 staining in tamoxifen-treated *Plp^+^* ^/^*^+^*;*Dnmt1^fl/fl^* and *Plp^creER(t)^* ^/^*^+^*;*Dnmt1^fl/fl^* NWM spinal cords. ***B***, Quantification of OLIG2^+^ and CC1^+^ cell densities in NWM^f^ (*p* = 0.3537, *p* = 0.3803). ***C***, Representative OLIG2 and CC1 staining in tamoxifen-treated *Plp^+^* ^/^*^+^*;*Dnmt3a^fl/fl^* and *Plp^creER(t)^* ^/^*^+^*;*Dnmt3a^fl/fl^* NWM spinal cords. ***D***, Quantification of OLIG2^+^ and CC1^+^ cell densities in NWM^g^ (*p* = 0.8926, *p* = 0.5109). ***E***, Representative OLIG2 and CC1 staining in tamoxifen-treated *Plp^+^* ^/^*^+^*;*Dnmt1^fl/fl^;Dnmt3a^fl/fl^* and *Plp^creER(t)^* ^/^*^+^*;*Dnmt1^fl/fl^;Dnmt3a^fl/fl^* NWM spinal cords. ***F***, Quantification of OLIG2^+^ and CC1^+^ cell densities in NWM^h^ (*p* = 0.3136, *p* = 0.2173). ***G***, Representative 5mC and OLIG2 stainings in tamoxifen-treated *Plp^creER(t)^* ^/^*^+^*;*Dnmt1^fl/fl^*, *Plp^creER(t)^* ^/^*^+^*;*Dnmt3a^fl/fl^*, and *Plp^creER(t)^* ^/^*^+^*;*Dnmt1^fl/fl^;Dnmt3a^fl/fl^* NWM spinal cords (white arrowheads indicate high-5mC^+^/OLIG2^+^ cells). ***H***, Quantification of low-, medium-, and high-5mC levels in OLIG2^+^ cells in tamoxifen-treated *Plp^+^* ^/^*^+^*;*Dnmt1^fl/fl^* and *Plp^creER(t)^* ^/^*^+^*;*Dnmt1^fl/fl^* NWM^j^. ***I***, Quantification of low-, medium-, and high-5mC levels in OLIG2^+^ cells in tamoxifen-treated *Plp^+^* ^/^*^+^*;*Dnmt3a^fl/fl^* and *Plp^creER(t)^* ^/^*^+^*;*Dnmt3a^fl/fl^* NWM^jj^. ***J***, Quantification of low-, medium-, and high-5mC levels in OLIG2^+^ cells in tamoxifen-treated *Plp^+^* ^/^*^+^*;*Dnmt1^fl/fl^*;*Dnmt3a^fl/fl^* and *Plp^creER(t)^* ^/^*^+^*;*Dnmt1^fl/fl^;Dnmt3a^fl/fl^* NWM^k^. Scale bar = 50 µm. Data are mean ± SEM. *n* = 4–6 animals, three sections per animal (Student’s *t test*, ANOVA).

We first quantified the number of OLIG2^+^ and CC1^+^ in NWM to address the effect of *Dnmt1* and/or *Dnmt3a* ablation itself on the generation of OPCs and OLs (Fig. 2*A*–*F*). We detected no difference in the number of OLIG2^+^ and CC1^+^ cells in any knock-out compared with control NWM (Fig. 2*B*, *D*, *F*). Moreover, there was no differences in 5mC expression levels in OLIG2^+^ cells in knock-out compared with control NWM (Fig. 2*G*–*J*).

**Figure 3.**
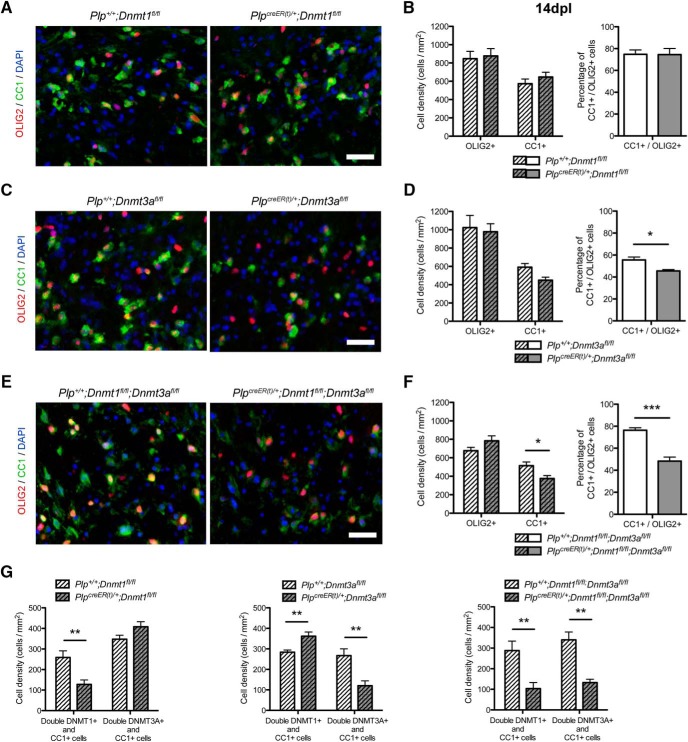
Ablation of *Dnmt3a* and both *Dnmt1* and *Dnmt3a* impairs oligodendrocyte differentiation during remyelination. ***A***, Representative OLIG2 and CC1 staining at 14 dpl in tamoxifen-treated *Plp^+^* ^/^*^+^*;*Dnmt1^fl/fl^* and *Plp^creER(t)^* ^/^*^+^*;*Dnmt1^fl/fl^* spinal cords. ***B***, Quantification of OLIG2^+^ and CC1^+^ cell densities and CC1^+^/OLIG2^+^ cells percentage at 14 dpl^l^ (*p* = 0.7955, *p* = 0.3573, *p* = 0.9689). ***C***, Representative OLIG2 and CC1 staining at 14 dpl in tamoxifen-treated *Plp^+^* ^/^*^+^*;*Dnmt3a^fl/fl^* and *Plp^creER(t)^* ^/^*^+^*;*Dnmt3a^fl/fl^* spinal cords. ***D***, Quantification of OLIG2^+^ and CC1^+^ cell densities and CC1^+^/OLIG2^+^ cells percentage at 14 dpl^m^ (*p* = 0.7851, *p* = 0.0550, *p* = 0.0149). ***E***, Representative OLIG2 and CC1 staining at 14 dpl in tamoxifen-treated *Plp^+^* ^/^*^+^*;*Dnmt1^fl/fl^;Dnmt3a^fl/fl^* and *Plp^creER(t)^* ^/^*^+^*;*Dnmt1^fl/fl^;Dnmt3a^fl/fl^* spinal cords. ***F***, Quantification of OLIG2^+^ and CC1^+^ cell densities and CC1^+^/OLIG2^+^ cells percentage at 14 dpl^n^ (*p* = 0.1510, *p* = 0.0357, *p* = 0.0006). ***G***, Quantification of DNMT1 and DNMT3A expression in CC1^+^ cells at 14 dpl in tamoxifen-treated *Plp^+^* ^/^*^+^*;*Dnmt1^fl/fl^* and *Plp^creER(t)^* ^/^*^+^*;*Dnmt1^fl/fl^*, *Plp^+^* ^/^*^+^*;*Dnmt3a^fl/fl^* and *Plp^creER(t)^* ^/^*^+^*;*Dnmt3a^fl/fl^*, *Plp^+^* ^/^*^+^*;*Dnmt1^fl/fl^;Dnmt3a^fl/fl^* and *Plp^creER(t)^* ^/^*^+^*;*Dnmt1^fl/fl^;Dnmt3a^fl/fl^* spinal cords, to detect eventual compensation between DNMTs at the protein level° (*p* = 0.0075, *p* = 0.0505, *p* = 0.0074, *p* = 0.0053, *p* = 0.0072, *p* = 0.0012). Scale bar = 20 µm. Data are mean ± SEM. *n* = 4–6 animals, three sections per animal. *p < 0.05, **p < 0.01, ***p < 0.001 (Student’s *t* test).

At 14 dpl, there was no difference in the number of OLIG2^+^ and CC1^+^ cells in the lesion or in the percentage of CC1^+^ differentiated OL among the OLIG2^+^ oligodendroglial cells between *Plp^+^* ^/^*^+^*;*Dnmt1^fl/fl^*controls and *Plp^creER(t)^* ^/^*^+^*;*Dnmt1^fl/fl^* mutants (Fig. 3*A*, *B*). Ablation of *Dnmt3a* resulted in a significant decrease of the percentage of CC1^+^ differentiated OL among the OLIG2^+^ oligodendroglial cells (Fig. 3*C*, *D*). This indicated that OPC differentiation was altered in mutants lacking *Dnmt3a*, whereas OLIG2^+^ proliferation and recruitment to the lesion was not affected (Fig. 3*D*). It was noteworthy that increased DNMT1 levels were detected in CC1^+^ cells in *Plp^creER(t)^* ^/^*^+^*;*Dnmt3a^fl/fl^* mutant spinal cords, suggesting that in the absence of DNMT3A there might be a compensatory increase in DNMT1 (Fig. 3*G*). To offset this possible effect, we performed a similar analysis on double knockout mice lacking both *Dnmt1* and *Dnmt3a*. Both the number of CC1^+^ cells in the lesion and the percentage of CC1^+^ differentiated OL among the OLIG2^+^ oligodendroglial cells were decreased in the double (*Plp^creER(t)^* ^/^*^+^*:*Dnmt1^fl/fl^;Dnmt3a^fl/fl^)* mutants, and to a greater extent than we observed in the *Dnmt3a*-only ablated (*Plp^creER(t)^* ^/^*^+^*;*Dnmt3a^fl/fl^)* mutants (Fig. 3*E*, *F*).

**Figure 4.**
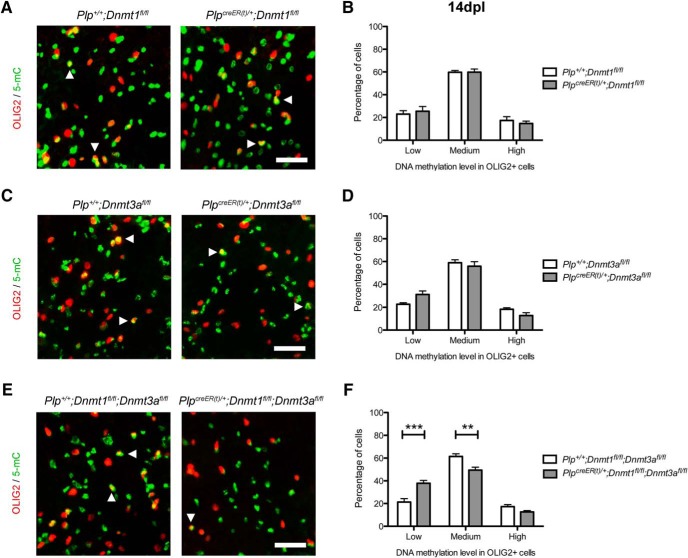
Ablation of *Dnmt3a* and both *Dnmt1* and *Dnmt3a* impairs methylation levels in oligodendroglial cells during remyelination. ***A***, Representative 5mC and OLIG2 staining at 14 dpl in tamoxifen-treated *Plp^+^* ^/^*^+^*;*Dnmt1^fl/fl^* and *Plp^creER(t)^* ^/^*^+^*;*Dnmt1^fl/fl^* spinal cords (white arrowheads indicate high-5mC^+^/OLIG2^+^ cells). ***B***, Quantification of low-, medium-, and high-5mC levels in OLIG2^+^ cells at 14dpl^p^. ***C***, Representative 5mC and OLIG2 staining at 14 dpl in tamoxifen-treated *Plp^+^* ^/^*^+^*;*Dnmt1^fl/fl^* and *Plp^creER(t)^* ^/^*^+^*;*Dnmt3a^fl/fl^* spinal cords (white arrowheads indicate high-5mC^+^/OLIG2^+^ cells). ***D***, Quantification of low-, medium-, and high-5mC levels in OLIG2^+^ cells at 14dpl^q^. ***E***, Representative 5mC and OLIG2 staining at 14 dpl in tamoxifen-treated *Plp^+^* ^/^*^+^*;*Dnmt1^fl/fl^* and *Plp^creER(t)^* ^/^*^+^*;*Dnmt1^fl/fl^;Dnmt3a^fl/fl^* spinal cords (white arrowheads indicate high-5mC^+^/OLIG2^+^ cells). ***F***, Quantification of low-, medium-, and high-5mC levels in OLIG2^+^ cells at 14 dpl^r^. Scale bar = 100 µm. Data are mean ± SEM. *n* = 4–6 animals, three sections per animal. **p < 0.01, ***p < 0.001 (ANOVA).

There were no changes in 5mC expression levels in OLIG2^+^ cells in *Plp^creER(t)^* ^/^*^+^*;*Dnmt1^fl/fl^* and *Plp^creER(t)^* ^/^*^+^*;*Dnmt3a^fl/fl^* mutants (Fig. 4*A*–*D*). However, there was an increase in the percentage of low-5mC–expressing OLIG2^+^ cells, associated with a decrease of medium-5mC–expressing OLIG2^+^ cells in the double *Plp^creER(t)^* ^/^*^+^*:*Dnmt1^fl/fl^;Dnmt3a^fl/fl^* mutants (Fig. 4*E*, *F*). This suggested an increase in low-methylated and a decrease in medium-methylated oligodendroglial cells in *Dnmt1/Dnmt3a* ablated mutants, which contrasted with the increased methylation previously observed in control animals (Fig. 1*H*, *I*).

**Figure 5.**
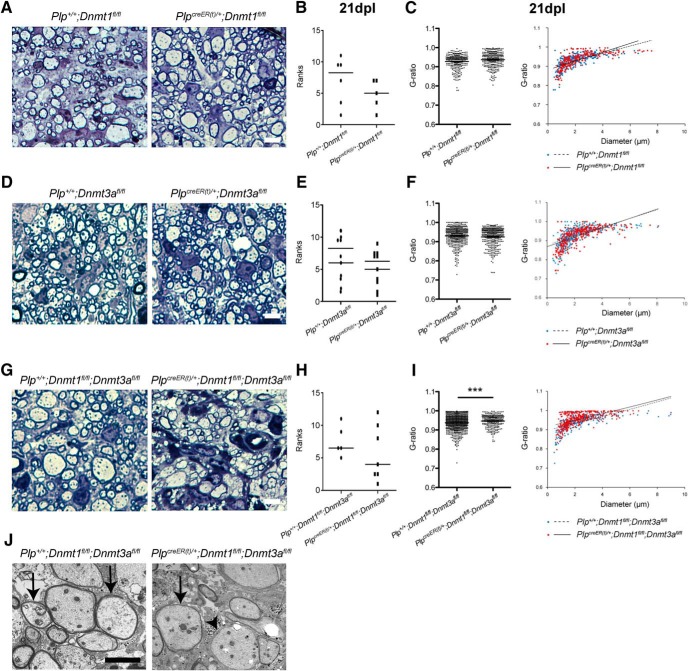
Ablation of *Dnmt1* and *Dnmt3a* impairs remyelination in the adult spinal cord. ***A***, Representative semithin sections at 21 dpl in tamoxifen-treated *Plp^+^* ^/^*^+^*;*Dnmt1^fl/fl^* and *Plp^creER(t)^* ^/^*^+^*;*Dnmt1^fl/fl^* spinal cords. ***B***, Relative ranking of remyelination^s^ (*p* = 0.3075). ***C***, Quantification of g-ratios for control and mutants mice, and plot of g-ratios against axonal diameter^t^ (*p* = 0.9426). ***D***, Representative semithin sections at 21 dpl in tamoxifen-treated *Plp^+^* ^/^*^+^*;*Dnmt3a^fl/fl^* and *Plp^creER(t)^* ^/^*^+^*;*Dnmt3a^fl/fl^* spinal cords. ***E***, Relative ranking of remyelination^u^ (*p* = 0.7144). ***F***, Quantification of g-ratios for control and mutants mice, and plot of g-ratios against axonal diameter^v^ (*p* = 0.1079). ***G***, Representative semithin sections at 21 dpl in tamoxifen-treated *Plp^+^* ^/^*^+^*;*Dnmt1^fl/fl^;Dnmt3a^fl/fl^* and *Plp^creER(t)^* ^/^*^+^*;*Dnmt1^fl/fl^;Dnmt3a^fl/fl^* spinal cords. ***H***, Relative ranking of remyelination^x^ (*p* = 0.7584). ***I***, Quantification of g-ratios for control and mutants mice, and plot of g-ratios against axonal diameter^y^ (*p* = 0.0005). ***J***, Representative electron microscopic sections at 21 dpl in tamoxifen-treated *Plp^+^* ^/^*^+^*;*Dnmt1^fl/fl^;Dnmt3a^fl/fl^* and *Plp^creER(t)^* ^/^*^+^*;*Dnmt1^fl/fl^;Dnmt3a^fl/fl^* spinal cords revealing new thin myelin sheaths of remyelination (arrows) and a demyelinated axon (arrowhead). Scale bar = 10 µm. Dots are ranking for each mouse (***B***, ***E***, ***H***) and g-ratio for each quantified axon (***C***, ***F***, ***I***). Data are mean ± SEM. *n* = 3–5 animals, >70 axons per animal. ***p < 0.01 (Mann–Whitney test and Student’s *t* test).

These data indicate a role for DNMT3A in adult OPC differentiation during remyelination that can be compensated for by DNMT1.

### Ablation of *Dnmt1* and *Dnmt3a* impairs remyelination in the adult spinal cord

To establish whether the impaired differentiation of OPC lacking DNMTs affected remyelination, we used a similar experimental design where we killed lesioned control and mutant mice at 21 dpl and evaluated remyelination by light microscopic examination of semithin resin sections stained with toluidine blue and by electron microscopy. Comparison of control and mutant NWM revealed no abnormalities in myelination in the three knockout mouse lines (data not shown). Ranking of remyelination on semithin sections (Fig. 3*A*, *B*, *D*, *E*) and quantification of the g-ratio (Fig. 3*C*, *F*) did not reveal any differences from controls for either *Dnmt1*- or *Dnmt3a*-ablated mice. In contrast, despite a similar ranking of remyelination in sections from controls and double mutants (both *Dnmt1* and *Dnmt3a* ablated; Fig. 3*G*, *H*, *J*), the quantification of g-ratio revealed thinner myelin in mutants, likely suggesting delayed remyelination in the absence of *Dnmt1* and *Dnmt3a* (Fig. 3*I*). This suggests that if *Dnmt3a*-only ablation is sufficient to reduce adult OPC differentiation in a lysolecithin-induced lesion, compensation by DNMT1 might prevent significantly delayed remyelination.

These data demonstrate that dysregulation of DNA methylation in adult oligodendroglial cells impairs their differentiation and hence their ability to contribute to remyelination.

## Discussion

Here we report that DNMT1 and DNMT3A are differentially expressed during remyelination after lysolecithin-induced demyelination in the adult spinal cord, with DNMT1 being highly expressed in OPCs at early time points after demyelination (corresponding in this model to the early stages of remyelination) and DNMT3A being highly expressed in OL at later time points (corresponding to the later stages and completion of remyelination). These data validate and extend previous microarray-generated data obtained in laser-capture microdissected tissues from rats with ethidium bromide–induced demyelinating lesions, which revealed initial increased expression of both *Dnmt1* and *Dnmt3a* and their subsequent decrease in expression (Huang et al. 2011). Discordance between the two studies, especially for DNMT3A expression, can be explained by differences in the experimental approach. The Huang et al. (2011) dataset was obtained from whole tissue, which has a mixed composition and percentage of various cell types at different time points, possibly impacting the levels of transcripts. Indeed, *Dnmt1* and *Dnmt3a* are also highly expressed by astrocytes and microglial cells, the latter being massively abundant in the lesion at 5 dpl but less abundant during later stages (Zhang et al. 2014).

Our study reports global hypermethylation in the nuclei of oligodendroglial lineage cells during remyelination, similar to what was described during developmental myelination (Moyon et al. 2016). These data suggested that adult OPC differentiation might recapitulate their developmental differentiation, by activating the same transcriptional pathways and perhaps the same epigenetic modulators (Fancy et al. 2004, 2009; Koenning et al. 2012; Nakatani et al. 2013; Moyon et al. 2015; Zhao et al. 2015). Indeed, chromatin remodelers (i.e., *Chd7* and *Brg1*) and histone deacetylases have been recently shown to be essential for OPC myelination as well as remyelination (Shen et al. 2008; He et al. 2016).

Using conditional knockout murine strains, we showed that lack of *Dnmt3a*, and not *Dnmt1*, in oligodendroglial cells impairs adult OPC differentiation. These data differed from the findings obtained during development, when the ablation of *Dnmt1*, and not *Dnmt3a*, resulted in extensive defective myelination of the CNS (Moyon et al. 2016). We also observed that, contrary to developmental data, loss of *Dnmt3a* was partially compensated for by upregulation of *Dnmt1* levels, leading to decreased adult OPC differentiation and remyelination delays in the double conditional knockout mice. It is important to highlight that the *Plp-creER(t)* line was used to target oligodendroglial lineage in an inducible manner in the adult spinal cord. Although PLP has been shown to be expressed in adult OPCs (Spassky et al. 1998; Ruffini et al. 2004; Lin et al. 2009), our ablation of *Dnmt1* and *Dnmt3a* may have targeted a more mature population, when cells have already exited the cell cycle, and thus when DNMT1 and DNMT3A might have a different impact. The remyelination delay observed here is also less drastic than the extensive and global hypomyelination affecting the *Olig1^cre/+^;Dnmt1^fl/fl^* mutant mice (Moyon et al. 2016). Adult OPCs tend to proliferate less than their neonatal counterparts, suggesting that the absence of DNMT1 may not as adversely affect their replication, cell division, and survival (Wolswijk and Noble, 1989; Wolswijk et al. 1991; Shi et al. 1998; Ruffini et al. 2004; Lin et al. 2009; Young et al. 2013; Moyon et al. 2015). Moreover, some epigenetic marks might have been already established and could be irreversibly maintained in adult OPCs, which are emerging from a pool of undifferentiating neonatal OPCs (Zawadzka et al. 2010). Indeed, it has been shown that in cell lines epigenetic markers such as histone methylation, histone deacetylation, and DNA methylation might have specific dynamics, with some being partial committers and others complete committers, depending on their enzyme recruitment speed and affinity at specific genomic sites (Bintu et al. 2016). For example, the *de novo* embryonic DNMT3B is a slow silencer but complete committer, as its methylated marks could not be easily removed. Thus, it could explain why ablation of *Dnmt3a* in adult OPCs might have a limited effect and only delay remyelination, as its markers would be maintained long after the enzyme ablation.

Finally, DNA methylation has been shown to be dysregulated in several neurologic pathologies, including amyotrophic lateral sclerosis, schizophrenia, and oligodendroglial pathologies such as MS and gliomas (Chou et al. 2012; Martin and Wong, 2013; Huynh et al. 2014; Hannon et al. 2016; Jaffe et al. 2016). In addition to neuropathy, dementia, and hearing loss, patients with DNA methyltransferase (*DNMT1*) mutations present with mild CNS hypomyelination (Klein et al. 2011). Epigenome-wide methylation study has identified several hypermethylated or hypomethylated loci in MS patient postmortem brain tissues compared with controls (Huynh et al. 2014) and several studies in gliomas have described an extensive global DNA hypomethylation (Watanabe and Maekawa, 2010; Chou et al. 2012) associated with site-specific DNA hypermethylation (Felsberg et al. 2006; Sharma et al. 2010). Further epigenome-wide studies should be performed on adult OPCs to specifically identify genomic loci that might be hypo- or hypermethylated during their proliferation and their differentiation, in control conditions and after demyelination or in gliomas. We propose that modulating DNA methylation in oligodendroglial cells could efficiently regulate adult OPC proliferation and differentiation capacities. Targeting DNA methylation at specific genomic loci, using engineered zinc fingers or CRISPR-Cas9 methylation modulators, might lead to the development of new therapeutic strategies in gliomas and MS (Choudhury et al. 2014; Heller et al. 2014; McDonald et al. 2016).

## Synthesis

Reviewing Editor: Jun Chen, University of Pittsburgh Decisions are customarily a result of the Reviewing Editor and the peer reviewers coming together and discussing their recommendations until a consensus is reached. When revisions are invited, a fact-based synthesis statement explaining their decision and outlining what is needed to prepare a revision will be listed below. The following reviewer(s) agreed to reveal their identity: Rehana Leak, Stephen Crocker.

Both reviewers agreed that your study is on an important topic, and well designed and well executed. The findings reported in this manuscript are novel and largely supported by the data. The use of DNMT1 and DNMT3 conditional knockout mice is another major strength of this study. Nevertheless, both reviewers had a number of concerns and suggestions, most of which should be straightforward to address and would help the authors to further improve the manuscript. Specific points by the reviewers are copied below:

Reviewer #1

There are a few concerns with this paper, as outlined below.

1. The authors show in Figure 2D that CC1+ cells are reduced in the DNMT3A knockouts by about 25-30%. This marker is shown in green in Figure 2C, but when I count individual green cells in the two panels in this figure, I observe no difference of this magnitude. Furthermore, it is not possible to estimate from the images the number of CC1+/OLIG2 cells in this figure, as I cannot see more than 1-2 double-labeled (yellow) cells and the overlap does not appear convincing (the quantification in panel D suggests the overlap should be 55% in the control animals). In contrast, the difference between the two panels in Figure 2E is convincing. Please show more convincing images for panel C of Figure 2.

2. There is no indication that the image analyses were performed by a blinded investigator. Without blinding, rigor is compromised. In general, the authors should add more descriptions that reveal the degree of rigor of this study. For example, more detail on the cell count methods would help readers reproduce the findings and judge the quality of the histological measurements. As written, the authors only write that the analysis was performed with Image J but with no further details, such as which anatomical subregion of the cord was focused upon, what size the images were, and the number of images captured per section. Did the authors simply use the threshold function in Image J or did they count the cells on the screen by eye with the manual Image J counting tool? A low power image of the region of interest in the context of the spinal cord would be useful for anatomical orientation.

3. In Figures 2 and 3, the authors do not show data from control animals that were not injected with lysolecithin. It is therefore not possible to estimate the fold changes relative to saline injections and compare this ratio across genotypes. This is important because transgene expression itself might change myelin thickness even in the absence of lysolecithin. Brief descriptions of previous work using the same exact model would mitigate this issue.

4. The authors conclude from Figure 3B that there is no difference in remyelination between the two groups, but given the apparent decrease shown in this figure and the small number of animals, this study seems to be underpowered. Can the authors comment on why there were only 3 mice in Figure 3B but 5 mice in Figure 3E? If the authors omitted any data points anywhere in the study, they should specify the exclusion criteria.

5. In Figure 3, the authors should include arrows that show loss of the myelin sheath in the double mutants on the EM images, as it is difficult to appreciate a difference between the two critical panels in Figure 3G. Higher magnification images would also be valuable here.

6. The authors argue on page 9 that the thinner myelin in the double mutants, as shown by the change in g-ratio, indicates “delayed remyelination” (line 193). However, there could be a number of other explanations for thinner myelination, such as a permanent disruption in the equilibrium between synthesis and breakdown. If the authors' sentence is indeed correct, please discuss in greater detail the evidence that myelination eventually recovers specifically in the double knockouts.

Reviewer #2

This is a very well written manuscript clearly describing the study hypothesis and experimental results. The approaches used to address the question were well described, straight-forward, appropriately executed and properly analyzed. There are some points for the authors to consider:

1. In the discussion the authors refer to a possible increase in DNMT1 in the DMNT3 cKO mice. While the authors do mention and provide data on 5-methylcytosine (5-mC) expression in Olig2+ cells in wildtype animals, there was no data on this epigenetic mark in any of the DNMT cKO lines studied. Analysis of 5-mC in Olig2+ cells in the DMNT1, DMNT3 and especially the double DMNT1 and 3 cKO mice should be provided.

2. There is no mention on whether loss of either DMNT1, DMNT3 or both together (without lesioning) had any impact on OPC numbers or CNS myelination. Any constitutive effect on OPCs and myelination resulting from gene expression loss alone should be taken into account.

3. G-ratio analyses of remyelination of axons is provided for all conditional knockout lines in Fig 3. The data provided do not provide information on the axon caliber, and thus, conveying these data as a scatter plot with axon diameter on the x-axis would provide additional, possibly salient, information on the effects of DMNT1 and DMNT3 in this injury model. Also, the ranking analyses was also curious in its ambiguity and relevance. The authors may consider additional analyses of other time points or excluding these data from the report.

4. The labeling of bars in the graphs, specifically the legends in Fig 1, stating for example “DMNT-1+ and NKX2.2” is not unambiguous. IF these are intended to represent double positive cells then the legends should reflect this as the current labels suggest that DMNT1+ cell counts may include non-Nkx2.2 cells. Clarification on this is required.
